# Global Consultation on Cancer Staging: promoting consistent understanding and use

**DOI:** 10.1038/s41571-019-0253-x

**Published:** 2019-08-06

**Authors:** James Brierley, Brian O’Sullivan, Hisao Asamura, David Byrd, Shao Hui Huang, Anne Lee, Marion Piñeros, Malcolm Mason, Fabio Y. Moraes, Wiebke Rösler, Brian Rous, Julie Torode, J. Han van Krieken, Mary Gospodarowicz

**Affiliations:** 1 Department of Radiation Oncology, Princess Margaret Cancer Centre, University of Toronto, Toronto, ON Canada; 2 Keio University School of Medicine, Tokyo, Japan; 3 Department of Surgery, University of Washington, Seattle, WA USA; 4 Department of Clinical Oncology, The University of Hong Kong and the University of Hong Kong–Shenzhen Hospital, Shenzhen, Guangdong China; 5 Cancer Surveillance Section, International Agency for Research on Cancer, Lyon, France; 6 School of Medicine, Cardiff University, Cardiff, UK; 7 Department of Oncology, Kingston Health Sciences Center, Queen’s University, Kingston, ON Canada; 8 Union for International Cancer Control (UICC), Geneva, Switzerland; 9 National Cancer Registration Service, London, UK; 10 Radboud University Medical Center, Nijmegen, Netherlands

**Keywords:** Epidemiology, Cancer screening, Neoplasm staging, Cancer models, Tumour biomarkers

## Abstract

Disease burden is the most important determinant of survival in patients with cancer. This domain, reflected by the cancer stage and codified using the tumour-node-metastasis (TNM) classification, is a fundamental determinant of prognosis. Accurate and consistent tumour classification is required for the development and use of treatment guidelines and to enable clinical research (including clinical trials), cancer surveillance and control. Furthermore, knowledge of the extent and stage of disease is frequently important in the context of translational studies. Attempts to include additional prognostic factors in staging classifications, in order to facilitate a more accurate determination of prognosis, are often made with a lack of knowledge and understanding and are one of the main causes of the inconsistent use of terms and definitions. This effect has resulted in uncertainty and confusion, thus limiting the utility of the TNM classification. In this Position paper, we provide a consensus on the optimal use and terminology for cancer staging that emerged from a consultation process involving representatives of several major international organizations involved in cancer classification. The consultation involved several steps: a focused literature review; a stakeholder survey; and a consultation meeting. This aim of this Position paper is to provide a consensus that should guide the use of staging terminology and secure the classification of anatomical disease extent as a distinct aspect of cancer classification.

## Abstract (web summary)

Attempts to incorporate additional criteria into the traditional tumour-node-metastasis staging classification have often resulted in inaccuracy and confusion in the use of terminology. In this Position paper, the authors provide guidance on the consistent use of the terminology relating to cancer staging.

## Introduction

Determination and documentation of the extent of disease in a patient is a prerequisite activity in starting the process of cancer care. For more than 70 years the anatomical extent of disease, or cancer stage, has been classified using the tumour-node-metastasis (TNM) classification, although, with evolving knowledge, many other factors have been recognized to also influence prognosis^1,2^. Some factors relate to patient characteristics (such as age and performance status), while others reflect more detailed tumour-specific elements that might reflect disease trajectory (such as hormone receptor status in women with breast cancer) or that might enable a more precise diagnosis (such as the presence of human papillomavirus DNA in patients with oropharyngeal cancer). Indeed, Pierre Denoix, who developed the original TNM classification, acknowledged that many factors other than the anatomical extent of disease can contribute to prognosis^3^. Unfortunately, attempts to include these additional factors in staging classification systems to date have led to increasing levels of miscommunication regarding the goals of cancer classification and variability in the application of such classifications in clinical research and practice and in cancer surveillance. Notably, cancer staging is not only used to define prognosis but is also applied to guide patient care; consistent staging is also vital in enabling cancer surveillance, in epidemiological studies and in the broader field of cancer control. Eventually, if an increasing variety of prognostic factors are included in future staging systems without careful adherence to logical and consistent principles, these classifications would no longer provide a distinct construct that accurately reflects the anatomical extent of disease. The exact description of disease burden might shift with refinements in methods of assessment, such as imaging, resulting in stage migration^4^. The availability and measurement of anatomical disease stage is more likely to be consistent over time and across different geographical areas compared with the ever changing field of molecular prognostic factors^5,6^. These other factors, biomarkers and measures of biological activity are more vulnerable to the many vagaries that can accumulate over time, including those emerging from new discoveries, assay development and clinical validation^5,7^. Furthermore, a potential biomarker might not be clinically relevant across the entire disease spectrum but might be highly effective in a specific anatomically defined subset of patients. For example, gene expression profiling provides valuable information on prognosis among patients with lymph node-positive (stage III) melanoma, but not in those with earlier stage disease^8,9^.

To clarify the objectives of cancer staging and promote its correct and consistent use, a Global Consultation on Cancer Staging was convened under the auspices of the Union for International Cancer Control (UICC), the US National Cancer Institute (NCI), and the Centers for Disease Control and Prevention (CDC) (Box 1). The aim of the Consultation was to review, discuss and promote the consistent understanding and use of cancer staging terminology.

Box 1 Participants in the Global Consensus Meeting

**Table 1:** 

Hisao Asamura	UICC TNM Atlas Co-Editor, IASLC
Fred Bosman	ICCR
James Brierley	UICC TNM Co-Chair
Robert K. Brookland	AJCC Vice-Chair
David Byrd	AJCC Chair
David Collingridge	*Lancet Oncology*
Meredith Giuliani	Educator
Peter Goldstraw	IASLC
Mary Gospodarowicz	UICC TNM Co-Chair
Frederick Greene	AJCC Past Chair
Shao Hui Huang	GCCS Secretariat
Peter Ingeholm	UICC Danish GAG Representative
Sean Kehoe	FIGO
Betsy Kohler	NAACCR
Carol Kosary	NCI
Ann Lee	UICC TNM GAG Co-ordinator
Malcolm Mason	UICC TNM Rapporteur
Fabio Moraes	UICC Young Leader
Serban Negoita	NCI
Brian O’Sullivan	UICC TNM Prognostic Factors Committee Chair
Max Parkin	IARC
Marion Piñeros	IARC
Brian Rouse	IARC
Zuzanna Tittenbrun	UICC
Julie Torode	UICC
Liesbet Van Eycken	UICC Education Coordinator, IACR
Han Van Krieken	UICC Netherlands GAG representative
Kevin C. Ward,	NCI
Hannah K. Weir	CDC
Christian Wittekind	UICC TNM Editor
Mei Ling Yap	UICC Young Leader

AJCC, American Joint Commission on Cancer; CDC, Centers for Disease Control and Prevention; FIGO, International Federation of Gynecology and Obstetrics; GAG, Global Advisory Group; GCCS, Global Consultation on Cancer Staging; IARC, International Agency for Research on Cancer; IASLC, International Association for the Study of Lung Cancer; ICCR, International Collaboration on Cancer Reporting; NAACCR, North American Association of Central Cancer Registries; NCI, National Cancer Institute; TNM, tumour-node-metastasis; UICC, Union for International Cancer Control.

## Development of cancer staging

The TNM system for the classification of disease extent was originally designed with the intention of quantifying the anatomical burden of cancer. Owing to the consistent, robust associations between anatomical extent of disease and patient outcomes, the TNM classification became synonymous with prognosis. The overarching importance of obvious, clinically apparent anatomical extent of disease to prognostication and clinical decision-making and the simplicity and ease of use of the TNM system led to the adoption of this classification system worldwide^1,10,11^. Over the ensuing years, several additional prognostic factors have occasionally been incorporated into staging classifications. Examples of these factors include patient age in the classification of differentiated thyroid cancers and histological grade in those designed for soft tissue and bone sarcomas, as well as the development of prognostic classifications for certain cancers, such as gestational trophoblastic neoplasms and lymphomas^11,12^.

The UICC, which has provided stewardship and enabled the development of the TNM classification from its origin onwards, also has a mandate to address the wide-ranging issues surrounding population-based cancer control, including the need to maintain a staging classification system that is relevant to an extensive group of stakeholders, such as the CDC, the International Agency for Research in Cancer (IARC), the International Association of Cancer Registries (IACR), and the International Cancer Benchmarking Partnership (ICBP). Appreciating that cancer registries are dependent upon consistent cancer staging and that many registries throughout the world have limited resources, the UICC collaborated with the IARC to develop the Essential TNM Classification, which is designed for use in resource-limited settings^12,13^. The UICC has also expended considerable effort in documenting and classifying the various nonanatomical prognostic factors that must be considered in clinical care^2^.

Since 1959, the American Joint Commission on Cancer (AJCC) has published its own TNM classification^14^. The AJCC was initially formed to evaluate the recommendations of the UICC TNM committee and to make suggestions or offer alternate classifications based on the TNM that were considered to be more suitable for use by physicians based in North America^15^. However, starting with the fourth edition in 1987, the aim is to ensure as little difference as possible between the two classifications^7,9^, and the UICC and AJCC strive to use congruent definitions, although the wording used in each version is not always exactly the same owing to differences in the traditional formalizations and styles used by the two organizations. With each new edition, some differences also arise, typically owing to topographical or formatting errors that have been corrected in respective websites and new printings. The AJCC has also made considerable efforts to improve the classification with the goal of enabling more accurate prediction of survival by including additional factors in its prognostic groups, which are also mirrored in the UICC prognostic groups discussed below.

In addition to the UICC and AJCC TNM classifications, other cancer staging systems exist and are used both for clinical and research purposes, including the International Federation of Gynecology and Obstetrics (FIGO) staging system, which is used for clinical, research and surveillance purposes, and the Surveillance, Epidemiology and End Results (SEER) Extent of Disease (EOD) classification, which is mainly used by the cancer surveillance community^16^. Members of the UICC TNM project also collaborate with the network of national and regional TNM committees, with the AJCC and FIGO, as well as the CDC, the World Health Organization (WHO) and the IARC to maintain a high level of uniformity between classification systems wherever possible^11,12,17^.

### Axes of cancer classification

Cancers are a heterogeneous group of diseases and are generally classified using the WHO International Classification of Diseases for Oncology (ICD-O), which includes topography and morphology codes^18^. The diseases are further characterized along two main lines — histological and molecular characteristics, and anatomical extent. Pathological classification criteria are outlined in the universally adopted WHO Classification of Tumours series (the so-called Blue Books), which provide an authoritative and concise resource for the histological and molecular classification of tumours. While recognizing these important paradigms, the anatomical extent of disease remains important and, in many instances, continues to be the most important determinant of patient outcome and is captured in the TNM staging classification.

### Treatment and prognosis

Cancer stage classification serves many purposes (Box 2), including patient care, prognostication and obtaining data to guide cancer control and research. A large constellation of factors influence prognosis, and these factors can relate to the tumour, the patient, and factors external to the patient or the patient’s environment, such as social determinants of health, including access and quality of healthcare (or treatment setting). These are important in the context of cancer management and, indeed, can affect all aspects of cancer control including clinical practice, research and surveillance, and the development, provision and maintenance of cancer systems and programmes. Some of these factors are remediable, such as access to quality care, while many are more difficult to ameliorate and often form part of the intrinsic characteristics of the tumour (such as tumour pathology). No uniformly accepted framework for classifying and/or integrating these factors into tumour staging currently exists, although attempts to incorporate one or more of these factors into staging classification frequently emerge^19–21^.

The UICC has maintained a traditional formalization that emphasizes staging based on anatomy, wherein the T, N, and M categories are combined into groups termed ‘stage’, with essential prognostic factors presented separately. In this way, the contribution of both the anatomical and nonanatomical domains can be differentially appreciated, thereby facilitating future revisions. In selected tumour histologies (such as thyroid cancers and sarcomas, as noted above) the UICC traditionally combined information on T, N, and M with other nonanatomical prognostic factors to maintain agreement with the AJCC (it was considered inappropriate to subsequently reverse this decision for these few cancers). The AJCC adopts a different approach, with a single grouping system applied following definition of the categories, referring to these as ‘prognostic stage groups’. When anatomical factors only are considered for the AJCC ‘prognostic stage groups’, these are identical to the UICC ‘stage’. In turn, the UICC ‘prognostic groups’ are identical to the AJCC ‘prognostic stage groups’ in the rarer situations in which nonanatomical factors are included in the UICC definition. In breast cancer, the AJCC provides cancer-specific prognostic stage groups that combine measures of the anatomical extent of disease with those of essential nonanatomical prognostic factors, while also retaining anatomical stage groups that consist of the anatomical extent of disease which is solely recommended for use in geographical regions in which the necessary biomarker tests are unavailable^11,12^.

Box 2 Objectives of cancer staging^12^

- To aid the clinician in the planning of treatment
- To give some indication of prognosis
- To assist in evaluation of the results of treatment
- To facilitate the exchange of information between treatment centres
- To contribute to the continuing investigation of human cancer
- To support cancer control activities

### Stakeholders

Multiple stakeholders rely on accurate cancer staging, potentially the most important being the patients themselves, in partnership with their physicians who require an indication of prognosis to make informed treatment decisions. Researchers, especially those involved in clinical trials, use information on staging to both select and stratify patients and to plan relevant clinical studies. Cancer registries and epidemiologists rely on accessible synoptic depictions of disease extent (including disease stage) across populations to understand patterns of presentation and differences in patient outcome, plan research projects, and study the effects of population-based screening and early detection programmes on disease stage at presentation^22–27^. Agencies with a role in cancer control also have an interest in disease stage at presentation, as this information can be used to guide resource allocation and to monitor compliance with treatment guidelines^12,25,28–30^.

The comparison of outcomes between jurisdictions and in a given population relies on the staging classifications and definitions being applied uniformly both across and within specific populations, and on consistent use of language and/or terminology. Indeed, inconsistencies in the classifications have hampered the comparisons of outcomes across geographical regions and nations as described by the ICBP and addressed in more detail below^31^. Thus, it is important for the classification to be consistent and to follow the published guidelines.

The requirement for staging systems that serve the needs of multiple stakeholders creates various challenges. Some stakeholders require classifications to be as simple and as stable over time as possible, while others prefer a more detailed classification that enables the rapid incorporation of new knowledge that is relevant to current clinical practice^5^. Thus, obtaining consensus through dialogue and agreement between different stakeholders remains a continued necessity.

## Consequences of inconsistent application

Inconsistent cancer staging can arise from misuse of the nomenclature. The potential for confusion exists in many areas, including the use of the T, N and M categories, the stage groups, the definitions of what constitutes clinical versus pathological extent of disease; and the definition of extent of disease at diagnosis versus following treatment. Adding to the confusion, communication regarding cancer stage between two or more populations can be hampered by inconsistent use of different staging classifications, use of different editions of the TNM classification and/or a lack of reporting of the specific classification and edition being used. The ICBP investigated disease stage at diagnosis and stage-specific survival using population-based cancer registry data obtained from six economically developed countries^31^. The study was hindered by a lack of information on disease stage (for example, availability was often limited to grouped TNM data, without reporting of the individual component T, N and M variables) and by substantial levels of variation in the type of stage classifications being used (including different editions of the TNM and/or the Dukes’ and FIGO systems). The investigators called for a global consensus to promote adherence to a single staging system^31^.

### Amalgamation of other prognostic factors

Considerable interest exists in the use of composite or mixed classifications that embody both disease extent and biology, owing to the importance of the additional prognostic factors. Such classifications can manifest as modifications to the T, N or M categories, or within the stage groups^32–34^. A tendency also exists to subdivide disease into ‘risk’ subgroups assembled using amalgams of different prognostic factors (for example, high-risk versus low-risk disease without identifying a specific outcome of interest or the components of the subgroups). Hybrid classifications of this type^32–34^ are typically fraught with the potential for confusion regarding understanding of the importance and relevance of different individual prognostic elements. In turn, this potential for confusion perturbs the purpose of staging as a classification reflecting the anatomical extent of disease.

We emphasize that the creation of risk groups or strata that are relevant to clinical practice is entirely feasible and appropriate once the fundamental components (including TNM categories, biological variables and others) are established. Composite risk or prognostic groups of this type are often helpful, although they also have limitations^35–37^. Such risk groups are usually not generalizable across the full spectrum of disease and tend to be specific to anatomy and time-dependent scenarios^21,38–40^. Furthermore, similar to any other classifications, the boundaries between the individual elements might be imprecise. For example, the levels of serum tumour biomarkers such as prostate-specific antigen and carcinoembryonic antigen are generally correlated with, but do not directly correspond to, the anatomical extent of disease. As a further example from prostate cancer, a high serum prostate-specific antigen level might reflect the extent of disease and predict a higher risk of distant metastasis, although a low level in the presence of known disease might be a consequence of poor differentiation^41^. Some previously accepted parameters, while posing challenges to the strict application of the rules of the TNM classification, might be accepted as historical exceptions, such as testicular teratoma. However, the existence of a few notable exceptions should not lead to the widespread incorporation of a wide range of nonanatomical variables into the TNM classification. Incorporating such variables increases the risk of not recognizing the unique contributions of both tumour biology and anatomical disease extent.

### The dynamic nature of prognosis

An awareness of the outcome being assessed, the specific intervention (whether that be observation or treatment), and the specific end point under consideration (whether that be 5-year survival, local tumour control, organ preservation, symptom control, or another end point) is important when determining a patient’s prognosis. The management and prognosis of a patient at the time of first presentation of disease is not usually the same as when a recurrence becomes apparent later; the consequences of recurrent disease might also differ appreciably depending on the location of the recurrence (locoregional compared with distant disease). These steps in the illness need to be considered separately because prognosis, prognostic factors, treatment options and outcomes are all likely to differ. In this way, the steps along the disease trajectory of a patient with cancer can be considered a series of scenarios. Even within certain scenarios, nuances might exist depending on how the first treatment evolves, and whether complications or additional problems emerge (such as the need for adjuvant treatment owing to adverse findings uncovered during initial surgery). Numerous scenarios in which both prognosis and prognostic factors can vary might exist for any patient during the course of their illness. In this way, prognosis can only be accurately defined in scenario-specific contexts (such as at initial presentation, at recurrence, or following the emergence of distant metastases) that include the effects of other prognostic factors and of the interventions administered at that time^42,43^.

## The consultation (planning and structure)

The UICC TNM Project, under the auspices of the NCI and the CDC, held the Global Consultation on Cancer Staging (GCCS) in 2017 to address the aforementioned issues of consistency and universal utility. The aim of this consultation was to reach agreement on the optimal use of cancer staging classification terminology and on stage definitions^44^ (Box 3). The discussions were limited to the role of the anatomical extent of cancer (cancer stage) and the terminology related to the stage, staging classification and the process of cancer staging. Other prognostic considerations relating to cancer biology, patient or host characteristics, and the environment that might influence quality of or access to care were deferred to a subsequent consultation designed to address this broader scope of prognosis and patient outcome.

An initial preparatory phase of the GCCS was undertaken to confirm the diverse use of staging terminology. A panel of international experts on the use of cancer staging was convened to investigate and evaluate the impressions of the UICC TNM Core Committee. The members of the UICC TNM Project, in consultation with the US NCI SEER Project and the AJCC, convened a planning group that also included representatives from the IARC, IACR, the International Collaboration on Cancer Reporting (ICCR) and the NCI. This group identified issues to be addressed and the appropriate interested stakeholders to survey. A survey of cancer clinicians and cancer registry community professionals was conducted to assess the current use of cancer stage terminology. Examples from the literature further confirmed the prevalent heterogeneous use of the concept of cancer staging and the related terminology.

A second phase included a face-to-face meeting of major stakeholders and users of cancer staging systems, in which current challenges to the consistent application of staging classification were presented and discussed in detail. The consultation focused on achieving agreement on how staging rules should be interpreted and applied.

### Survey on the use of terminology

An online survey was initially piloted among 10 independent experts. The survey was then refined for clarification of language and content and distributed to 463 TNM users worldwide who were randomly selected from a database of email addresses provided by several major TNM user groups: the UICC TNM Core Group; the UICC Expert Panels and Global Advisory Groups; the UICC Manual of Clinical Oncology authors; SEER Directors and Managers; The Canadian Council of Cancer Registries; and the IACR and AJCC Editorial Board and Expert Panels. In total, 143 (31% of selected TNM users) responded. The survey comprised 35 questions focusing on four different domains: application of the TNM classification; cancer stage terminology; prognostic factors; and prognostic classifications.

Analysis of the survey data revealed that 87.5% of respondents believed that the application of the TNM staging terminology is inconsistent in the literature. A large majority (85% of respondents) confirmed that multiple stakeholders use data on tumour stage; these include clinicians and patients when estimating prognosis and selecting treatments, researchers to facilitate clinical trial eligibility and stratification as well as when undertaking translational studies, and cancer control professionals when exploring cancer behaviour. A large majority (85% of respondents) also believed that individual T, N, and M categories, in addition to information on roman numeral stage grouping, should be recorded in both national and regional cancer registries. In addition to the TNM, 71% of respondents reported that information on other prognostic factors should also be collected by cancer registries, although 85% stipulated that information on the anatomical extent of disease should be collected as a separate and distinct variable. Many respondents (81%) reported that tumour biomarkers and other prognostic factors are important independent determinants of prognosis, but also that no overarching framework for classifying such factors can be applied for all cancers. No consensus emerged on how the TNM and other prognostic factors could be combined. Regarding cancer staging terminology, the majority of the respondents confirmed the view that the effects of stage migration are similar to the Will Rogers phenomenon, whereby changes in staging criteria can lead to spurious improvements in prognosis at certain disease stages, but most believed that stage shift is the same as stage migration^4^. The majority of respondents (61%) defined tumour downstaging rather than downsizing as a reduction in the size of a tumour after neoadjuvant therapy. In addition, no clear definition of the term ‘understaging’ emerged. The answers to the final section of the survey on terminology formed the basis of a list of definitions that were revised and confirmed at the Global Consultation Conference (BOXES 4–6).

### Inconsistent use of staging terminology

To better appreciate the level of inconsistency in the use and meaning of terms employed in cancer staging classification, pathological classification, biomarkers, tumour profile and prognostic group definitions, we conducted a limited review of the literature involving all articles published in any of 12 selected high-impact oncology journals between July and December 2016. The journals were screened for published articles (clinical trials, prospective and retrospective studies and review articles) pertaining to cancer staging, prognostic factors, prognostic groups and tumour pathology and/or profile classification. Inconsistent understanding and use of stage terminology was found in 20% of the reviewed literature (Box 7). Incorrect definitions of cancer stage and TNM categories were the most prevalent inconsistencies, with a variety of nonstandard terms such as ‘T stage’, ‘T group’, ‘T status’ and others being used instead of the officially designated terms ‘T category’ or ‘TNM stage’. Furthermore, somewhat random use of the terms discussed in the survey results was also identified; ‘downsizing’, ‘upsizing’ and ‘understaging’ were all evident and applied in different scenarios without clear explanations or definitions. ‘Downstaging’ and ‘downstage’ were used both as a noun and verb and were defined inconsistently. Similarly, the terms ‘stage shift’ and ‘stage migration’ were defined in multiple ways and applied inconsistently. The published literature also included information on post-treatment factors (such as the status of tumour resection margins), together with baseline pretreatment factors, when reporting the clinical TNM stage. Information on tumour biomarkers was frequently combined with information on the anatomical extent of disease. Furthermore, in some articles^38^, temporally distinct patient populations were combined into a single population, such as the inclusion of information on the characteristics of patients with primary and those with recurrent tumours within the same group.

### The Consultation Meeting

The GCCS meeting was held in London, UK. Experts from the UICC, AJCC, NCI, CDC, FIGO, IACR, IARC and the ICCR participated. A general agreement was reached regarding the observed challenges and the needs of the consultation as detailed above, and the details of the literature review and the survey results were analysed to fully appreciate the extent of variability in the application of the T, N, M and cancer staging terminology. The participants reaffirmed the purpose of the staging classification, discussed the process of staging and the importance of applying it within the appropriate clinical scenario, recognized the scope of TNM staging, provided guidance for its appropriate use, and discussed and agreed upon the definitions of relevant terms (BOXES 4–6). A unanimous consensus was reached that anatomical extent of disease and other prognostic factors should be considered and presented separately. Lessons learned through the GCCS will be adopted and implemented in the next iteration of the TNM classification, the TNM ninth Edition.

Box 3 Process and methods: the Global Consultation on Cancer Staging Planning phase: a panel of international experts on cancer staging was convened

- To confirm the need for a consultation
- To plan the consultation

Literature review:

- To identify inconsistencies in the use of cancer staging classification terminologies
- Target literature reviewed included English language items published in 12 high-impact journals between July 2016 and December 2016.

A stakeholder survey was undertaken to evaluate the current understanding and use of terminology surrounding cancer stage classification

- 35 questions were prepared following a pilot study
- Addressed application of TNM, cancer stage terminology, prognostic factors and prognostic classifications

The Global Consultation on Cancer Staging

- To engage experts on cancer staging, global health, cancer care and clinical research
- To discuss inconsistencies in the use of cancer staging terminologies
- To develop consensus on cancer staging terminologies

TNM, tumour-node-metastasis.

Box 4 Fundamental definitions **Cancer stage**

- Should be defined and recorded at initial diagnosis, and must remain unchanged in the medical records.
- While anatomical disease extent may change during patient management, the original stage designation should not be amended.

**T category, N category, M category**

The anatomical extent of disease is based on the assessment of three components: the T category, the N category and the M category and should not be termed T stage, N  stage, and M stage because the term ‘stage’ is ordinarily reserved for the roman numeral aggregated stage groups.

**‘Clinical’ and ‘Pathological’ classification**

- **‘Clinical classification’ (cTNM):** the pretreatment clinical classification is based on evidence acquired before any treatment intervention. Such evidence is gathered from physical examination, imaging, endoscopy, surgical exploration and other sources.
- **‘Pathological classification’ (pTNM):** based on evidence acquired before treatment but supplemented or modified by additional information acquired from observations during surgery and from pathological examination of surgical specimen(s). This therefore requires knowledge of both the clinical and pathological extent of disease and cannot be solely determined by the pathologist without full clinical information or input.

TNM, tumour-node-metastasis.

Box 5 Stage concepts *Stage migration* describes a change in the proportion of T, N or M categories among patients within a defined population, following the introduction of a new means of assessing the extent of disease.

*Stage shift* describes a change in the pattern of stage distribution among patients within a defined population to a lower disease stage following the introduction of early detection or screening programmes, or to a higher disease stage when access to care becomes limited.

*Clinical TNM categories* (and, when available, pathological categories) should be recorded at diagnosis in cancer registries and used to determine clinical and pathological stage groups. Occasionally, when incomplete information is available in a cancer registry, the pathological and clinical data have been combined in order to avoid losing information; the consequence otherwise is an inability to assign a stage group. In such instances, the resultant stage group is neither a clinical nor pathological stage group but a combined version; the term *harmonized stage* (hTNM) has been proposed for such situations to facilitate cancer surveillance. In cancer care, all the variables pertaining to the extent of disease should be available to the clinician and the term ‘harmonized stage’ should not be used.

*Stage* is applied by the UICC to describe the anatomical extent of disease.

*Prognostic group* is applied by the UICC to describe classifications that incorporate prognostic factors in addition to the anatomical extent of disease.

*Anatomical stage group* is applied by the AJCC to classify breast cancers in geographical regions in which the required biomarker tests are not readily available to describe the anatomical extent of disease.

*Prognostic stage group* is generally used by the AJCC to describe groups that include anatomical extent of disease when combined with other prognostic factors, although in many situations the term is limited to the inclusion of anatomical disease extent if additional prognostic factors are not included.

*Surveillance, Epidemiology, and End Results Extent of Disease (SEER–EOD)* is a data coding system used by the surveillance community, but not generally by clinicians, that applies the terms limited, regional and distant to describe the anatomical extent of disease. This system is not straightforward to translate into the TNM system owing to substantial differences in the definitions of regional involvement.

AJCC, American Joint Commission on Cancer; TNM, tumour-node-metastasis; UICC, Union for International Cancer Control.

Box 6 Scenario-specific contexts *Restaging* is the process of investigating the anatomical extent of disease after initial treatment or management. All ‘scenarios’ other than initial diagnosis involve restaging and require different terminology in order to achieve clarity. A prefix should be used as follows:

- After neoadjuvant therapy — ‘*y stage*’
- After a documented disease-free period (complete response) and later recurrence, mandating ‘restaging’ — ‘*r stage*’.
- Anatomical extent of disease after a period of surveillance (as observed in prostate or thyroid cancer) — ‘*r stage*’

*Downstaging* describes a reduction in T (tumour) or N (node) category after neoadjuvant therapy

*Downsizing* describes a reduction in tumour volume after neoadjuvant therapy

The terms ‘*upstaging*’ and ‘*understaging*’ are occasionally used in the literature, and typically relate to differences in diagnostic accuracy of various staging investigations. We do not recommend the use of these terms owing to a lack of precision in understanding their meaning.

Box 7 Inconsistancies and incorrect use of terminology

- Incorrect use of T, N and M categories and TNM grouped stage, T or N status, group, subgroup and/or substage
- Misunderstanding of downstaging, downsizing and the definitions of other specific terms
- Misunderstanding the definition of stage shift
- Misunderstanding the definition of stage migration
- Attempting to define and assess prognosis in mixed patient populations, including combining those with primary and recurrent, recurrent and metastatic, or resectable and unresectable disease
- Mixing measures of nonbaseline factors and baseline factors in prognostic models
- Mixing tumour profile characteristics and cancer stage classifications to define prognosis

TNM, tumour-node-metastasis

## Next steps

The most important definitions and usage conventions relating to the TNM classification should be disseminated further to ensure consistent application and use among practitioners, researchers and cancer registry personnel. Both the introduction section of the UICC TNM Classification and Chapter 1 of the AJCC Cancer Staging Manual describe the rules and conventions of cancer staging using the TNM classification. Existing educational tools such as the UICC Helpdesk and the introductory site-specific modules and short educational videos are available on the UICC website, and educational webinars and clinical staging cases (staging moments) relating to chapter 1 of the AJCC manual are available on the AJCC website and require better promotion.

The diverse needs of various stakeholders should be better addressed. Cancer registry data should include a record of disease stage at initial presentation. A cancer registry would not normally classify a different disease extent as a criterion for disease progression during a period of ‘watchful waiting’ or ‘active surveillance’ (which is a frequently used approach in the management of patients with prostate cancer); by contrast, a clinician would be interested in the anatomical extent of disease at the time of definitive treatment and would usually document the degree of disease progression as the patient migrated into a new clinical scenario in this manner. The TNM classification rules state that the new disease extent should be classified using the ‘r’ prefix to reflect recurrent disease if progression occurred (BOX 6). However, this is not the current practice and merits the introduction of an agreed convention. The application of the concept of ‘cancer scenario’, as discussed previously, is therefore encouraged in order to improve the consistency of reporting.

Consistency and completeness of reporting would be enhanced by applying checklists to facilitate the verification of the proper use of terminology by journal editors and reviewers, for which compliance would be required. This action would be similar to the 2005 guidelines to improve the quality of reporting of study results, such as the Reporting Recommendations for Tumour Marker Prognostic Studies (REMARK)^45^ and those for prediction models involving Transparent Reporting for Individual Prognosis or Diagnosis (TRIPOD)^46^.

In the future, methodologies that enable the identification and inclusion of necessary data elements relevant for personalized treatment need to be explored. The UICC has developed prognostic factor tabulations that stratify these factors according to their relevance to patient management as determined by their inclusion in published cancer treatment guidelines. This process should be further optimized by collaboration with the established national and international evidence-based guideline development groups, such as the UK National Institute for Health and Care Excellence (NICE), the National Comprehensive Cancer Network (NCCN), European Society for Medical Oncology (ESMO), the International Consortium for Health Outcomes Measurement (ICHOM) and others, in order to ensure optimal alignment, consistency and value in cancer management strategies. The selection of relevant factors should pay particular attention to their inclusion in cancer registries where appropriate.

A consensus emerged from the GCCS meeting that the anatomical extent of disease and other prognostic factors should be considered separately, but could still be combined in the framework as separate ‘prognostic groups’ where applicable. The UICC TNM group publish breast cancer-specific stage and stage groups based on anatomical extent of disease and also separately publish essential prognostic factors required for treatment decision-making^12^. By contrast, the AJCC publishes a prognostic classification of breast cancer based on anatomical extent of disease combined with essential nonanatomical prognostic factors (oestrogen receptor, progesterone receptor and human epidermal growth factor receptor 2 status, and tumour grade)^11^. However, the AJCC also supports the aforementioned use of stage groups in the classification of breast cancers based solely on anatomical factors in geographical regions where quantification of the necessary biomarkers is not possible^11^.

More formal collaborations with international organizations involved in overseeing standards in cancer research and treatment should be encouraged and emphasized, and should particularly involve the IARC and WHO, in order to reduce inconsistencies in terminology and classifications between those described in the WHO Blue Books and the UICC^12^/AJCC^11^ classifications. In this regard, formal collaborations between the UICC, AJCC and WHO already exist. In addition to the selection and inclusion of relevant nonanatomical factors in addressing prognosis, the focus needs to include appropriate scientific methodologies for analysis of more complex emerging data, which are often voluminous and difficult to manage. Such approaches require rigour in developing predictive models and consultation with and contributions from experts in such analyses.

## Conclusions

The management of patients with cancer requires consistent terminology to define and accurately describe diagnosis, guide patient management and consider prognosis. Accordingly, cancer surveillance requires consistent definitions and indications to describe disease stage at diagnosis at the population level. Diagnosis is generally described using universally accepted ICD and ICD-O categories and the WHO Classification of Tumours. The TNM Classification of Malignant Tumours^12^ remains the standard for recording and classifying anatomical disease extent. The TNM Classification is neither designed nor equipped to encompass the entire spectrum of prognostic and predictive variables in all forms of cancer and further progress in developing distinct prognostic classifications or tools for improved estimation of prognosis will be needed in the future. The TNM Classification is important for many stakeholders with an interest in cancer control; therefore, adherence to precise definitions and using the terminology as designed to optimize the accuracy of clinical and scientific communications should be a priority. The GCCS has achieved a consensus on purpose and definition, in a variety of applications of cancer staging. This consultation also reaffirmed the need to promote education on the use of cancer staging, investigate the issue of cancer prognosis and develop methods to more accurately describe and calculate the probability of relevant outcomes in patients with cancer.
